# Structural Evolution of Hydrothermally Derived Reduced Graphene Oxide

**DOI:** 10.1038/s41598-018-25194-1

**Published:** 2018-05-01

**Authors:** Hsin-Hui Huang, K. Kanishka H. De Silva, G. R. A. Kumara, Masamichi Yoshimura

**Affiliations:** 1 Graduate School of Engineering, Toyota Technological Institute, Nagoya, 468-8511 Japan; 2 National Institute of Fundamental Studies, Kandy, 20000 Sri Lanka

## Abstract

Hydrothermal reduction is a promising approach for graphene oxide (GO) reduction since it is environmentally friendly, simple, and cost effective. We present a detailed study of structural changes occurring in graphene oxide during the reduction process. The correlations between the interlayer spacing, chemical states, work functions, surface morphology, level of disorders, the number of layers, and processing time are elucidated. The results reveal that GO flakes remain in the early stage of the reduction process and that they are fully reduced after a 4-h hydrothermal treatment. With an increase in the reduction time, the resulting product, *i.e*., reduced graphene oxide, has a low oxygen content, small interlayer spacing, and crumbled and wrinkled structures. We are convinced that these properties can be tuned to a desired level for various applications.

## Introduction

Graphene, a two-dimensional monolayer of carbon atoms in a hexagonal lattice with an sp^2^ bonding hybridization, has come to the forefront in the field of materials science and nanotechnology since the early 2000 s in view of its outstanding electrical and thermal properties combined with excellent mechanical strength^1–3^. These superior properties lead to graphene making a significant impact on the field of materials science and nanotechnology, with graphene now being considered to replace other materials used in existing applications. To date, the most common methods used to fabricate graphene are micromechanical exfoliation, chemical vapour deposition, and chemical oxidation and reduction of graphite^3–5^. However, each of these methods has certain problems and limitations, *e.g*., in terms of yields, defect contents, costs, steps, or production time^6^. Chemical oxidation and reduction of exfoliated graphite is the best solution among all the other methods due to the relative ease of creating sufficient quantities of products at a desired quality level. However, the chemical reduction involves the use of hazardous reducing agents, such as hydrazine or sulfonate, and its residues might have a significant effect on the structures and properties of the final products. On the other hand, hydrothermal reduction is a simple, fast, and environmentally friendly route which involves water only. The experimental setup is rather cost effective and easy and it only requires an autoclave with a Teflon-lined container along with a furnace. It has been reported that a closed system with certain temperature and internal pressure promotes the restoration of the aromatic structure which is favourable for minimizing the defects^7^. Moreover, it has a good scalability and suits industrial large-scale production.

Hydrothermal process was first introduced in the late nineteenth century, and it was mainly used in the production of synthetic minerals^8,9^. Since then, many studies have been performed across a wide range of the field, and the attention on the process is still growing. For instance, a graphene/silicon composite can be hydrothermally pruced for use as an anode in lithium ion batteries. A hydrothermally fabricated molybdenum disulphide (MoS_2_)/graphene composite shows a good onset potential in the hydrogen evolution reaction, giving one of the best performances among MoS_2_-based catalysts^10,11^. Reduced graphene oxide (rGO) decorated with titanium dioxide synthesized via a hydrothermal approach exhibits improved photocatalytic properties and would, therefore, be a promising material for future photovoltaic applications^12^. Additionally, composites of graphene and V_2_O_5_ have been developed for enhanced electrochemical energy storage^13^. Recently, a new form of self-assembled hydrogel rGO developed through a hydrothermal route has received a great deal of attention owing to the high mechanical strength (storage modulus of 450–490 kPa) of 1–3 orders of magnitude higher than conventional self-assembled hydrogels^14^, high compressive strength of 6 orders higher than conventional graphite products^15^, and an electrical conductivity as high as 5 × 10^−3^ S/cm^14^. Moreover, a three-dimensional Ni_x_Co_1-x_S_2_ particle/graphene composite hydrogel was shown to have an interconnected porous network with pore sizes in the range of several micrometres giving high performance as the active material in supercapacitors^16^.

While hydrothermal treatment is a unique synthetic approach to graphene oxide reduction and even though it has been used for years, it is faced with the challenges of firmly understanding the deoxygenation activity, preparing graphene with high quality and dispersibility, and precisely controlling the structure and morphology. Moreover, the structures and properties of the resulting products might vary depending on the control parameters used in the reduction process. Thus, it is necessary to clarify the reduction mechanisms and structural changes throughout the process, since the structure is strongly related to the final properties and hence the performance of the devices. Here, we present a detailed study on the structural, morphological, and electrical changes during the reduction process from the initial 30 min up to 10 h at 200 °C; hereafter, we refer to the individual samples as rGO 0.5 h, 1 h, 2 h, 4 h, 8 h, and 10 h. The gradual changes in the morphology and dramatic drop in the interlayer spacing were elucidated using X-ray diffraction (XRD), scanning electron microscopy (SEM), atomic force microscopy (AFM), and transmission electron microscopy (TEM). The degree of oxidation and reduction and the defects such as vacancies were confirmed using Raman spectroscopy and X-ray photoelectron spectroscopy (XPS). The electrical properties of the reduced graphene oxide at different stages were revealed through investigation of the strong correlation between oxygen content and contact potential difference/work function. We provide an insight into the structural evolution during graphene oxide reduction through a controllable hydrothermal route. We greatly believe that the resulting products can be tailored, according to their specific properties and structures, for desired applications.

## Results and Discussion

**Figure 1:**
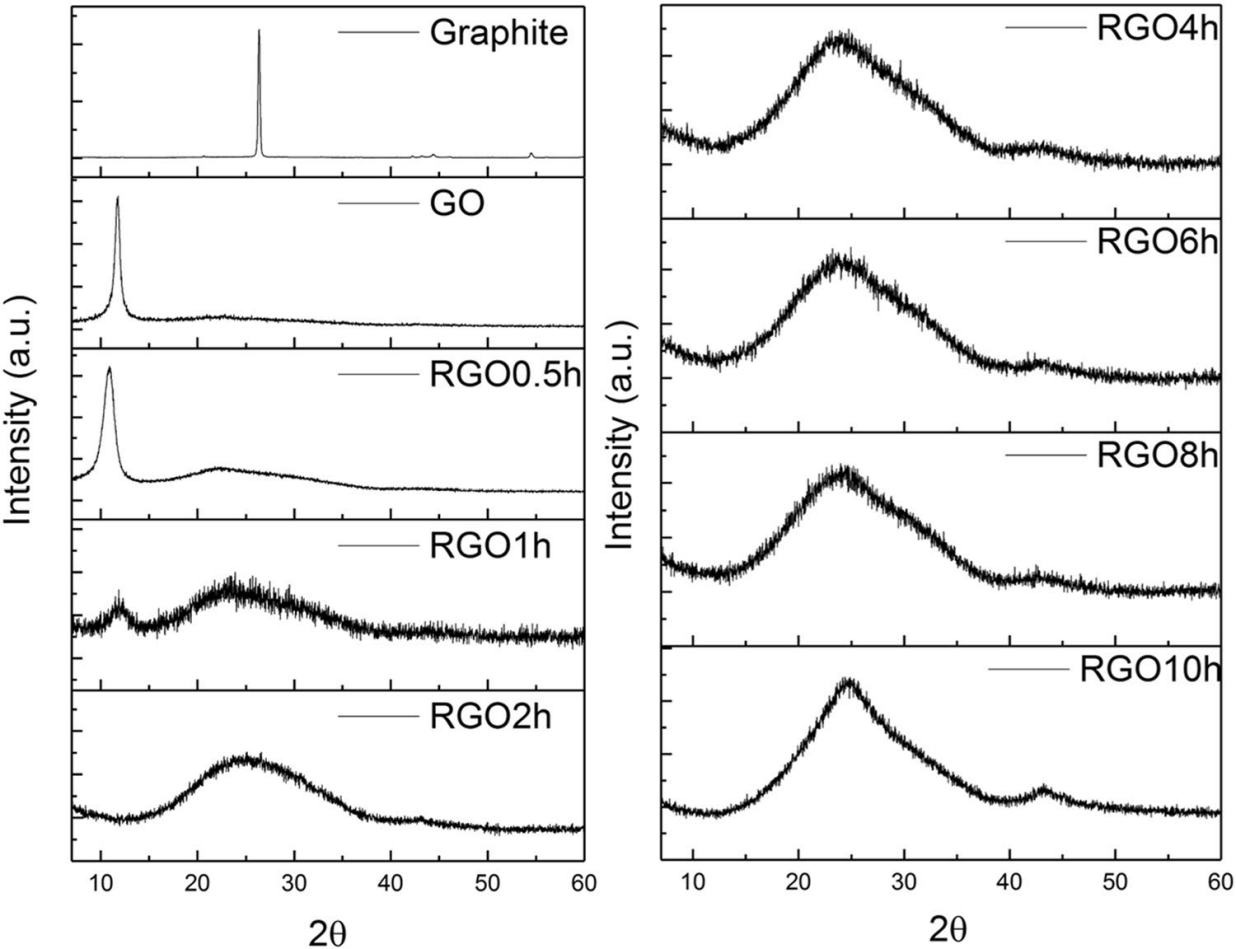
XRD patterns of graphite, GO, and rGO samples treated at different hours.

The structural change in the GO flakes was first revealed by XRD analysis, as shown in Fig. 1. In raw graphite, an intense crystalline peak was found at 2θ = 26.4° (lattice spacing of 0.34 nm), which corresponds to the (002) diffraction peak of graphite^17^. After oxidation, the peak shifts to a lower angle at 2θ = 10.9° with a lattice spacing of 0.81 nm, indicating the success in oxidation. As reported earlier, an increase in interlayer spacing is mainly attributed to the intercalation with water and the presence of oxygen functionalities such as epoxide and hydroxyl groups which populate on the basal plane of the carbon sheet^18–21^. Notably, the sharp and distinct peak is attributed to the preserved and ordered stacking along the c-axis. After 30-min hydrothermal treatment, the intensity of the distinct peak in GO reduces and the peak becomes broader due to the partial breakdown of the long-range order of the GO. Moreover, the peak position has slightly shifted towards a higher angle (2θ = 11.1°) showing the decrease in the lattice spacing (0.80 nm). Meanwhile, a relatively weak and broad shoulder was observed at 2θ = 22.8°, yielding an interlayer separation of 0.39 nm. This indicates that the reduction has taken place within 30 mins. After a 1-h hydrothermal reduction, the broad and diffuse peak becomes dominant. The interlayer spacing was calculated to be the (002) graphite peak of 0.35 nm. However, a weak GO peak still can be observed (2θ = 11.9°, d = 0.75 nm), denoting the coexistence of rGO and GO or incompletion of GO reduction. After 2 h reduction, the GO peak was undetectable and left a broad rGO peak on the spectrum with a d-spacing of 0.35 nm. The rGO peak position remains almost the same up to a reduction time of 10 h (details are shown in the Supporting Information Table S1). Intriguingly, in sample rGO 10 h, the intensity of the rGO peak increases corresponding to restacking or overlapping of rGO sheets^22^.

**Figure 2:**
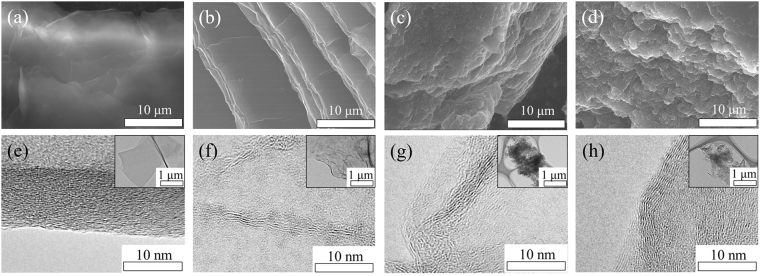
SEM images of (**a**) GO, and its deoxygenated samples treated under (**b**) 1 h, (**c**) 4 h and (**d**) 10 h. The corresponding TEM images are shown in (**e**–**h**), respectively. The insets of TEM images showing the sheets in low magnification.

**Figure 3:**
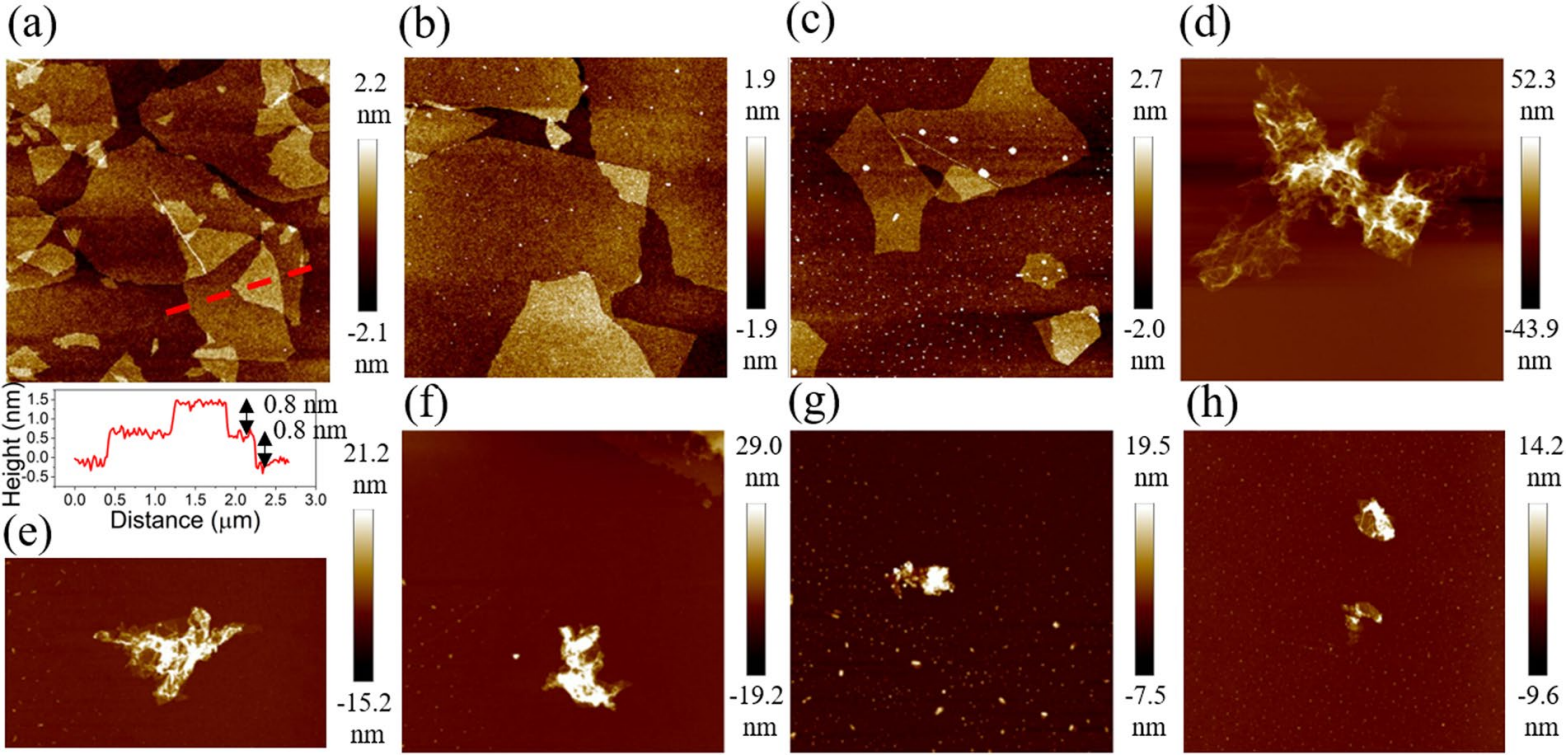
AFM images of the rGO samples treated at (**a**) GO, rGO (**b**) 30 min, (**c**) 1 h, (**d**) 2 h, (**e**) 4 h, (**f**) 6 h, (**g**) 8 h, (**h**) 10 h.

The surface morphology of GO, and hydrothermally reduced GO were analysed by SEM, TEM and AFM. Before hydrothermal treatment, the GO sample (Fig. 2(a)) shows a laminated structure. A monolayer or a few-layer GO could be achieved after dispersing in water, in which the thickness of a single layer was measured to be 0.8–0.9 nm, as observed by TEM and AFM (Figs 2(e) and 3(a)). The charging effect seen in the image has arisen due to the non-conductive nature of GO resulted from the defects and oxygen functionalities. Further, SEM and TEM images of the rGO 1 h, 4 h and 10 h are shown in Fig. 2(b–d) and (f–h) (SEM and TEM images of rest of the rGO samples can be found in the Supplementary Informations S1–S3). SEM image of rGO 1 h shows that the sheets still possess the layered structure. However, the TEM image of rGO 1 h sample indicates the coexistence of GO (layered structure) and rGO (disordered structure). Meanwhile, the number of layers reduces to less than 10 (Fig. 2(f)) showing the breakdown of the long-range-order stacking, which is consistent with XRD observation. With the increase in reduction time, the layered nature becomes disordered, crumpled and smaller. This behaviour can be visualized in the AFM topographic image in Fig. 3. It shows that the rGO flakes are flat, similar to the GO flakes, when they are treated for less than or equal to 1 h. According to the XRD results, a small GO peak appears in rGO 1 h sample but vanishes when reduction time increases to 2 h. It implies that the rGO 0.5 h and 1 h samples might still preserve the nature of GO giving the applicability to make a uniform thin film or membrane for related applications. In contrast, rGO 2–10 h samples show many wrinkles. The possible explanation is; in GO dispersion, the presence of negatively charged oxygen functionalities provides static repulsive force stabilizing the exfoliated sheets. Thus, when they are dropped onto the substrate, monolayer GO sheets lie flat on the substrate. On the contrary, when GO sheets are reduced, they become regionally hydrophobic due to the removal of the oxygen functionalities, as confirmed by XRD and X-ray photoelectron spectroscopy results (discuss later). After dropping onto the substrate, the locally hydrophobic rGO sheets will tend to aggregate to reduce the free energy, leading to the formation of wrinkled and folded morphology. Moreover, the edge-to-edge attraction interactions due to the hydrogen bonding between the remaining oxygen functionality might result in the aggregation of rGO sheets.

**Figure 4:**
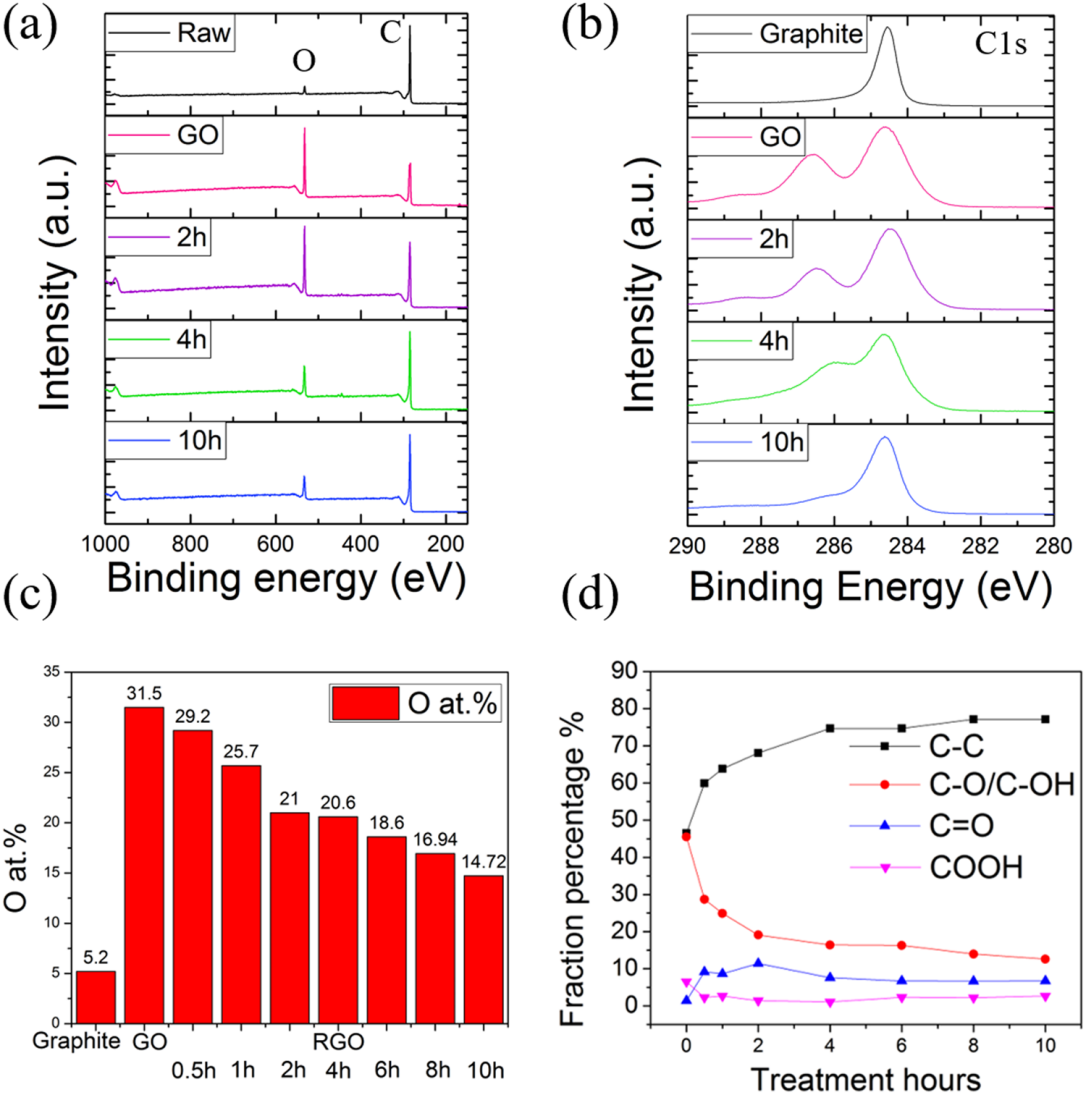
(**a**) XPS full spectra of raw graphite, GO, rGO 2 h, 4 h, and 10 h samples and (**b**) the corresponding high resolution C1s spectra. (**c**) Oxygen content as a function of reduction time. (**d**) Change in fraction percentage of the individual oxygen function group with reduction time.

In order to probe the chemical structure, we performed XPS on raw graphite, GO, and the series of rGO samples; the full-scan spectra, higher resolution C1s spectra, atomic percentage of oxygen, and fraction percentage of each oxygen functionality are shown in Fig. 4(a–d), respectively (full analyses of all the specimens can be found in the Supporting Information Figure S4). Initially, pristine graphite contains a small amount of oxygen of 5.2 at% (from the atmosphere or trapped in the Earth’s crust) which yields a high carbon to oxygen (C/O) ratio of 16.9. After oxidation, a well-defined double peak with a small tail towards higher binding energy was found in the high resolution C1s spectrum, which is a signature of a considerable degree of oxidation. It is also demonstrated by a significant increase in oxygen contents to 31.5 at%. It is noted that the C peak in sample GO showed a clear shift towards higher binding energy, reflecting significant surface charging caused by the electrically insulating oxygen functional groups. Here, it has been shifted back for ease of analysis. The C1s spectrum of GO was deconvoluted into four major components, including C-C/C=C (284.6 eV), epoxide C-O/hydroxyl C-OH (286.1–287.1 eV), carbonyl C=O (288.2 eV), and carboxyl COOH (289.3 eV) groups. Due to the similar binding energies, it is challenging to precisely determine a separate quantification of epoxide and hydroxyl groups, and therefore, their XPS peaks are discussed together.

Upon 0.5 h reduction (Figure S4), the double peak is present but there is a small decrease in the oxygen-related peak. The fraction of the epoxide/hydroxyl groups drops from 46% to approximately 34%. Based on the XRD analysis, the intensity of the GO peak is greatly reduced and, meanwhile, a small broad peak corresponding to rGO appears indicating that the GO has been partially reduced and converted to rGO. This suggests that the reduction of GO took place in such a short period of time due to the removal of thermally unstable epoxy groups^23^. After 2 h, the fractional percentage of C-O/C-OH bonds was further reduced but not fully eliminated. According to the density functional theory calculations reported by Kim *et al*.^24^ and Gao *et al*.^25^, the hydroxyl group attached to the edge of the sheets and the lattice vacancies is more stable. Therefore, the peak which is contributed to C-O/C-OH groups still can be seen. Interestingly, the rGO 2 h sample has a lattice parameter corresponding to that of graphite/graphene with a relatively small oxygen content, giving it an enhanced hydrophilicity (contact angle is approximately 58.6°), as shown in the Supporting Information Figure S5.

**Figure 5:**
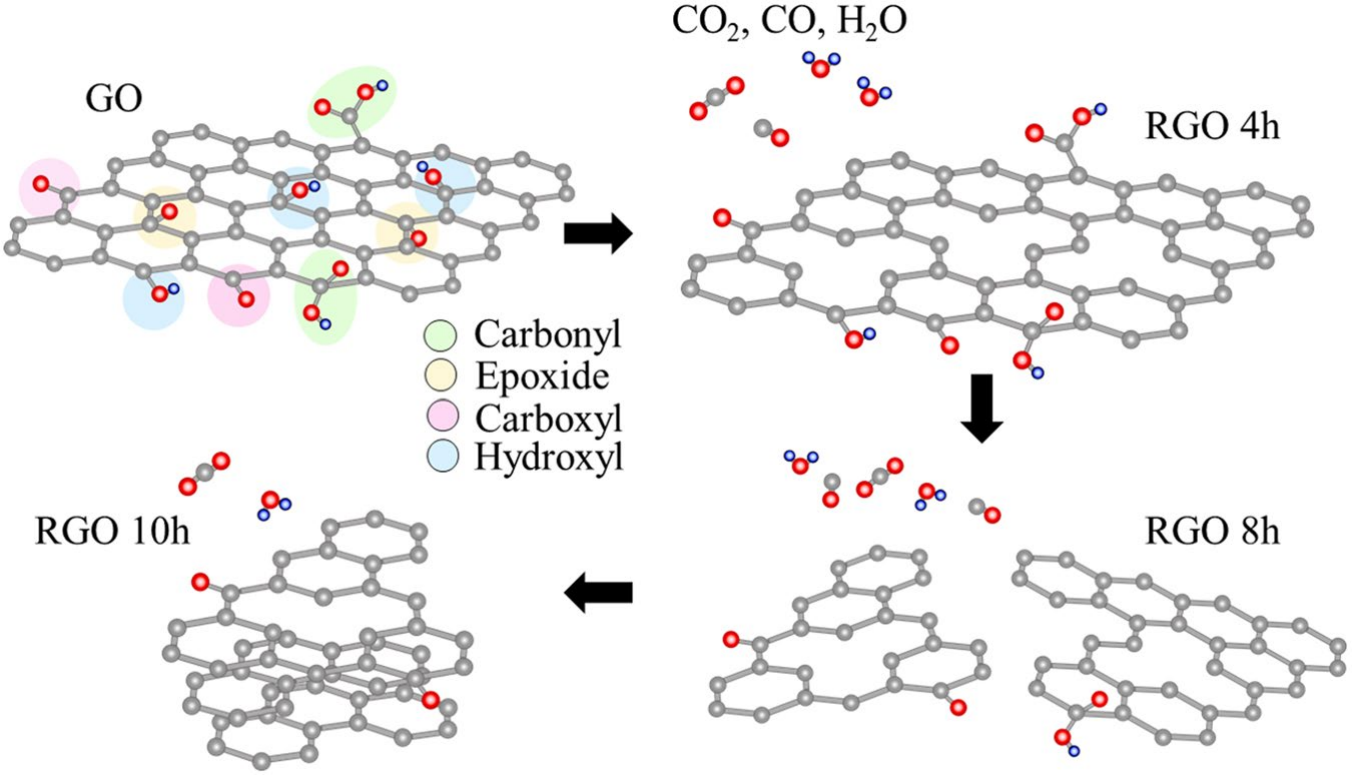
Schematic illustration of GO reduction with time dependence.

Upon further hydrothermal reduction, several changes were observed. The intensity of the C1s gradually increases while the intensity of the O1s peak drops with increasing reduction time, as shown in Fig. 4(a) and (c). It is noted that the C-C bond is stable during the treatment, whereas the C bonded to oxygen-carrying functionalities decreases which results in an increase in the C-C (sp^2^ and sp^3^) component contents. The oxygen content reaches the lowest value of 14.7 at% for sample rGO 10 h; at this point, the C-O/C-OH fractional content also decreases to its minimum value. This suggests that the C-OH bond is the major residual oxygen functional group which is the key metric to determine the level of reduction. Additionally, when the C-OH and C-O groups are removed, they break/cut off the rGO sheets into small pieces; as a consequence, the sheets will agglomerate to reduce the surface energy and re-stacking could occur, as shown previously and illustrated in Fig. 5. The success in GO reduction and the evidence of aggregation in the 10 h sample are further demonstrated by ultraviolet (UV)-visible spectroscopy (Supporting Information Figure S6). Ma *et al*. have reported similar observations: the large sheets (>2 μm) are almost absent after hydrothermal treatment and that the size of the rGO is dependent on the hydrothermal treatment temperature^26^. In brief, this implies that deoxygenation could be regulated by the hydrothermal reduction time. It is worth noting that only C and O peaks were observed in the spectra for all the samples, indicating that no other undesired compounds were formed due to the use of water as a reductant.

**Figure 6:**
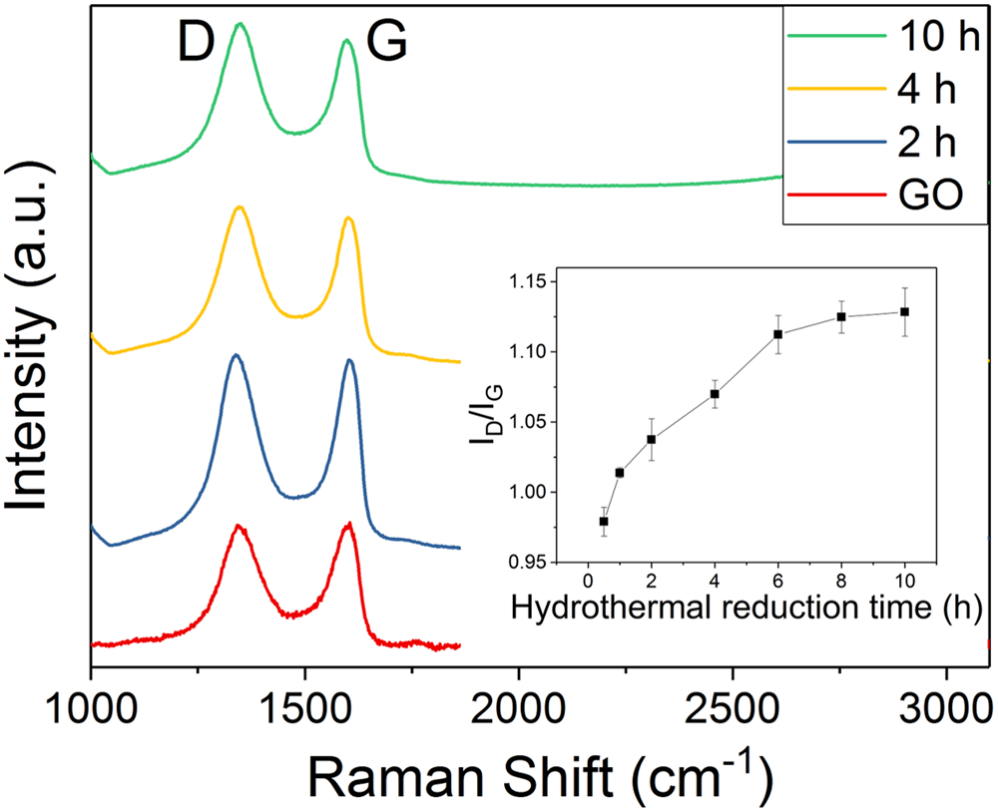
Raman spectra of graphene oxide, rGO 2 h, 4 h, and 10 h samples. (**b**) Insert shows the intensity ratio of D band over G band for GO and rGO treated at different hours.

Raman spectroscopy offers further insights to the structural changes in rGO (Fig. 6). The main features of the GO and its derivatives have D, G, and 2D peaks. The G peak at around 1580–1600 cm^−1^ is due to the bond stretching of sp^2^ carbons in rings and chains, while the D peak at approximately 1330–1340 cm^−1^ originates from the breathing modes of the six-membered rings that are activated by defects^27^. The change in the intensity ratio of D and G bands (I_D_/I_G_) with time-dependent GO reduction is plotted in the inset of Fig. 6. It shows a slight yet gradual increment of the I_D_/I_G_ ratio with the increase in reduction time. In the early stage of reduction, the I_D_/I_G_ ratio slightly increases which refers to the increase in edge of the sheets when sp^2^ domains are torn apart by the removal of the oxygen functionalities^28^. With further reduction, deoxygenation continues as demonstrated in XPS results, which might remove some of the carbons from the graphitic structure and leave pores and defects. Hence, it results in a deteriorated structure, giving an increase in the I_D_/I_G_ ratio. However, one should be noted that a solid conclusion is tough to make in the interpretation of Raman spectra for GO and its derivatives. This is due to the manifold defects within the carbon lattice which makes almost no change in the Raman spectra as compared to the pristine graphene or single-layer or few-layer GO and rGO^29^. The Raman spectra of all the samples are shown in the Supporting Information Figure S8.

**Figure 7:**
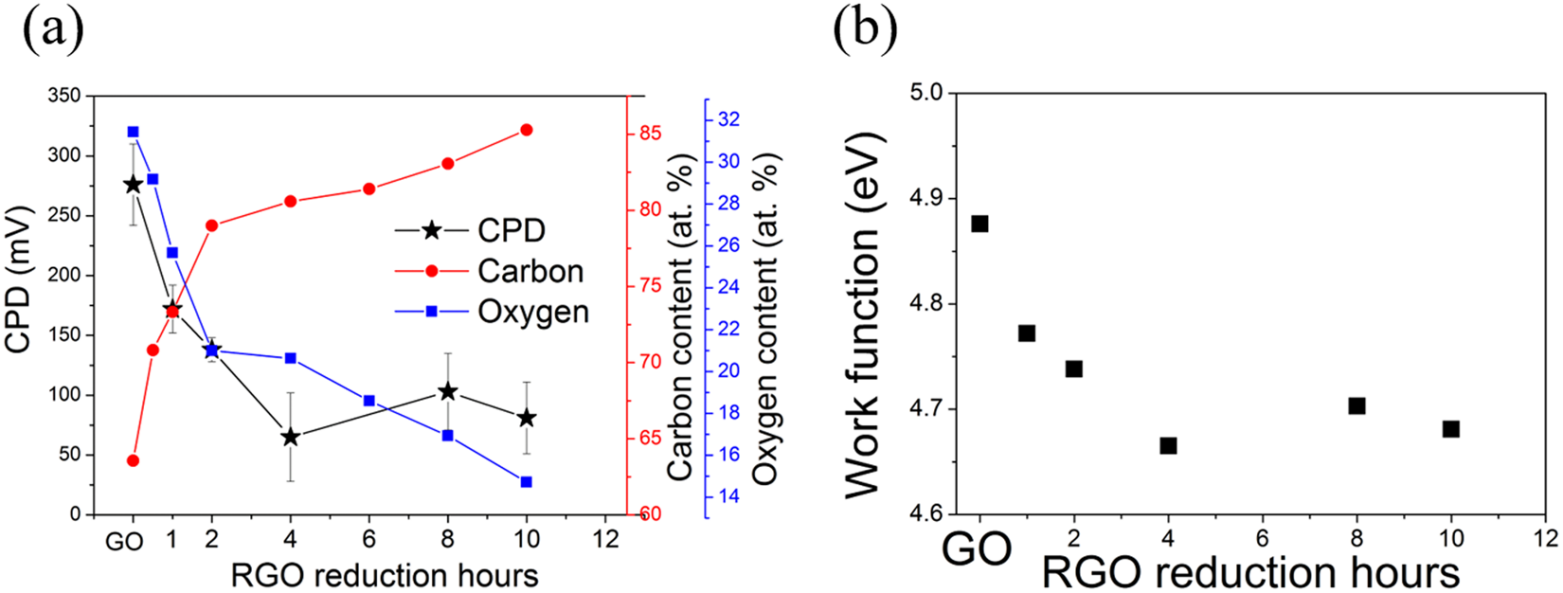
(**a**) The changes in CPD, carbon and oxygen content of rGO samples with its associated time dependence. (**b**) The work function of rGO samples as a function of reduction time.

The electronic properties of materials can be probed through the work function (WF), which can be derived from the contact potential difference (CPD) measured using Kelvin probe force microscopy (KPFM). Basically, the KPFM image maps the variation of the surface potential on the sample. By using a substrate with a known work function, the work function of the sheet can be calculated from the measured CPD difference (ΔV_CPD_) between the substrate and sheet, as shown in the following equation, ΔV_CPD_ = 1/e (Φ_substrate_ − Φ_film_), where Φ denotes the work function^30^. The measured V_CPD_ of GO and rGO samples were plotted along with carbon and oxygen content in Fig. 7(a). Initially, GO has the highest V_CPD_ which might be attributed to the large amount of chemisorbed oxygen on the graphene surface. During the GO reduction process, the CPD value decreases gradually with time. It is seen that the profile of oxygen content is similar to that of the CPD curve, while the carbon content shows the opposite trend. As reported earlier, the work function of a material is dependent on the chemical potential (such as oxidation state and defect sites) and surface dipoles^31–33^. In GO, the oxygen atoms, such as hydroxyl and epoxide, are bonded with carbon on either side of the basal planes with specific dipole moments. The strength of the dipole moment can be altered by modifying the overall oxygen content during reduction; thus, it is expected to lead to changes in CPD/work function. Even though here we show that a similar trend of work function and oxygen content were observed, which implies a clear correlation between the two, oxygen content might be merely one of multiple parameters including temperature, thickness, specific oxygen functionalities, *etc*. that contributes to the work function. More specifically, a theoretical study with molecular dynamics and density functional theory calculations has reported that each oxygen-containing functional group has different impact on the work function^34^. Nevertheless, at this stage, we considered only the correspondence of total oxygen atomic percentage with CPD changes.

The calculated work functions of all the samples are plotted in Fig. 7(b). It has been shown that the electronic properties can be enhanced by deoxygenation of GO. Care must be taken because the measured CPD is not simply due to the WF of the sheets but also due to the more complicated interplays between the electrical properties of the GO or rGO, underlying substrates, surface properties like atom absorption, the type of tip, *etc*.^35^. Therefore, the measured WF should be considered as the work function of the aggregate systems. Here, we mainly compared the trend and the changes in WF/CPD for series rGO samples under the same measurement conditions, such as, the use of the same tip and substrate. A more systematic study focused on, among other things, the effects of interfacial dipoles and substrates will be the subject of a future investigation.

**Figure 8:**
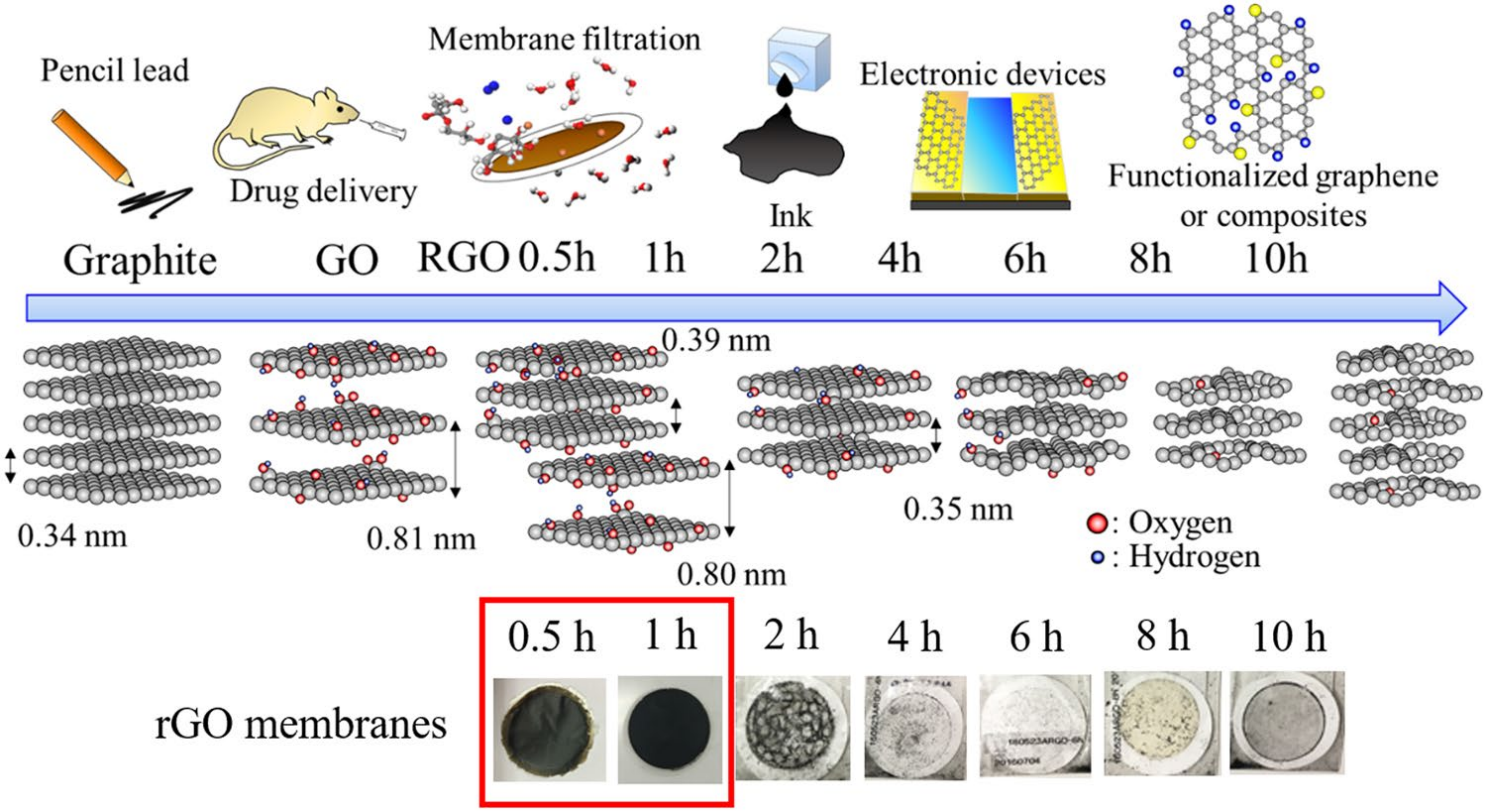
Schematic illustration of overall structural evolution of hydrothermally reduced GO and its potential applications. Lower part: the photographs of rGO membranes formed at different time intervals.

Overall, the structural evolution from raw graphite to oxidized graphite to reduced graphene oxide along with its associated time dependence is summarized in the schematic in Fig. 8. Understanding the structural and microstructural changes during the reduction process offers valuable information in terms of its applications, since the structure of the material is strongly related to its physical, electronic, and mechanical properties, and hence the performance in applications. In this work, we observed that GO and rGO coexist in sample rGO 0.5 h and 1 h preserving the lattice spacing of graphite and high hydrophilicity similar to GO phases. The rGO membrane can be formed under this condition (see Fig. 8), which can be utilized in the field of water desalination and purification where the interlayer spacing is a key factor for ion molecular sieving^36,37^. Additionally, it could possibly be applied to ink production since it possesses high dispersity due to the presence of oxygen functional groups and acceptable electronic properties. Samples rGO 2 h to rGO 8 h have better electronic properties due to the removal of the oxygen functional groups. The damaged and porous rGO 10 h sample provides the sites for polymerization or metal oxide decoration/doping. In conclusion, we can design or tune the structure of the materials by controlling or modifying the parameters in the hydrothermal reduction process to satisfy the requirements for future or current devices. A few potential or existing applications is schematically illustrated in Fig. 8.

## Methods

### Synthesis of graphite oxide

GO was synthesized by modified Hummers’ method^5^. 1 g of graphite powers with average particle size of 7 µm (Sri Lanka natural graphite, RS Mines) was used as a starting material. Oxidation of graphite was achieved by treating with concentrated sulfuric acid and potassium permanganate, followed by adding the hydrogen peroxide. Lastly, the graphite oxide dispersion was washed with 1 M hydrochloric acid solution and distilled water with the assistance of centrifuge to remove the excessive soluble ions until it reaches neutral pH value. Noted that the lower deposited gel-like dark-brown product was collected and dried in vacuum for hydrothermal reduction.

### Fabrication of reduced graphite oxide

A liquidous dispersion of GO was prepared by adding the dried GO flakes into the distilled (DI) water (1 mg/ml). It was then transferred to a Teflon-lined autoclave (HU-50, San-Ai Kagaku Co., LTD.), and heated in an electric furnace under 200 °C (NHK-170AF, Nitto Kagaku Co., LTD.). Samples were extracted from the furnace at different hours, 0.5 h, 1 h, 2 h, 4 h, 6 h, 8 h, and 10 h. The resulting product appeared in black and hereafter we named the individual samples as rGO 0.5 h, 1 h, 2 h, 4 h, 8 h, and 10 h. The rGO 0.5 h and 1 h were found to have high dispersity as the dispersion have no aggregates, while the black precipitates were found sinking at the bottom of the container for the rest of the samples. The suspensions were then vacuum filtered and dried at room temperature for further characterization.

### Characterization

Atomic force microscopy (AFM, Bruker MultiMode 8) and field-emission scanning electron microscopy (FE-SEM, Hitachi S-4700) were used for structural and morphological analysis. GO sample was observed using an accelerating voltage of 5 kV to eliminate the charging effect, while the rGO samples were viewed at an accelerating voltage of 15 kV. Interlayer spacing was determined by X-ray diffraction (XRD, Rigaku-Ultima IV X-ray diffractometer) with a step size of 0.02°/min using a CuKα1 radiation (λ = 0.154 nm). The elemental composition analysis was performed by X-ray photoelectron spectroscopy (XPS, ULVAC-PHI PHI5000 Versa Probe II) with a source of AlKα 1486.6 eV. Transmission electron microscopy (TEM, JEOL JEM-2100) operated at an accelerating voltage of 200 kV was used to study the microstructure and morphology of the samples. The TEM specimen was prepared by dispersing powders in ethanol and followed by ultrasonication for 15 mins. It was then dropped on the carbon film-coated copper grids for observation. Few-layered GO and rGO samples were used for Raman measurements. The GO or rGO dispersions were first dropped on a Si substrate with a layer of 300 nm-thick SiO_2_ followed by spin coating. Raman spectroscopy was conducted (Renishaw - InVia Raman Spectroscope) using a 532-nm laser. It is noted that each sample was measured for more than 10 points and Raman spectra were taken in the central areas of the flakes. Electronic property was examined by a frequency-modulated Kelvin probe force microscopy (FM-KPFM) (MultiMode 8, Bruker) in a peakforce tapping mode. A platinum-coated silicon cantilever (Olympus, OMCL-AC240TM-R3) was used. UV-visible spectra were collected using a V-650 spectrophotometer (Jasco) in the wavelength range of 200–500 nm with a resolution of 2.0 nm. Noted that all the dispersions were prepared at the same concentration, however, rGO 10 h sample could not be dissolved completely due to the high hydrophobicity in nature.

## Electronic supplementary material

Supplementary Information
