# Supplementary material for: Structural Evolution of Hydrothermally Derived Reduced Graphene Oxide
Source: Sci Rep. 2018 May 1;8:6849. doi: 10.1038/s41598-018-25194-1 (PMC5931577; doi:10.1038/s41598-018-25194-1)
Supplement: Supplementary file 1 — Supplementary Information [file 41598_2018_25194_MOESM1_ESM.docx]

**Supporting Information**

**Structural Evolution of Hydrothermally Derived Reduced Graphene Oxide**

Hsin-Hui Huang^1^, Kanishka De Silva^1^, G.R.A. Kumara^2^, Masamichi Yoshimura^1*^

^1^Graduate School of Engineering, Toyota Technological Institute, Nagoya, 468-8511, Japan

^2^Department of Chemistry, University of Peradeniya, Peradeniya, 20400, Sri Lanka

*Corresponding author. Tel: +81 52-809-1851. E-mail: [yoshi@toyota-ti.ac.jp](mailto:yoshi@toyota-ti.ac.jp) (Masamichi Yoshimura)


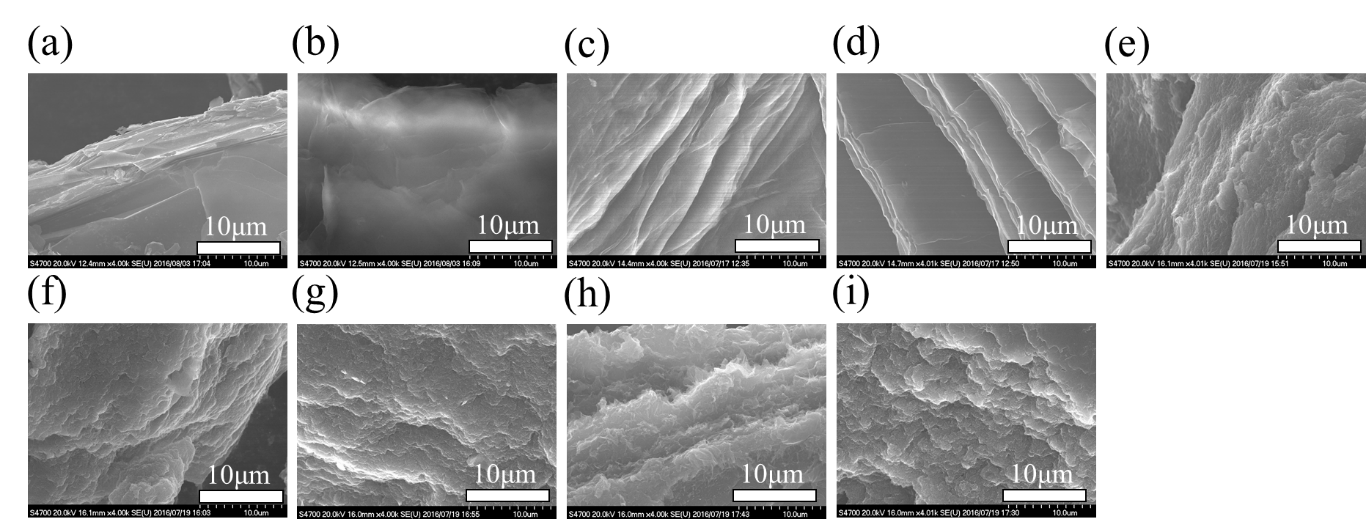


Figure S1 SEM images of (a) graphite, (b) graphene oxide, rGO (c) 0.5 h, (d) 1 h, (e) 2 h, (f) 4 h, (g) 6 h, (h) 8 h, and (i)10 h samples.


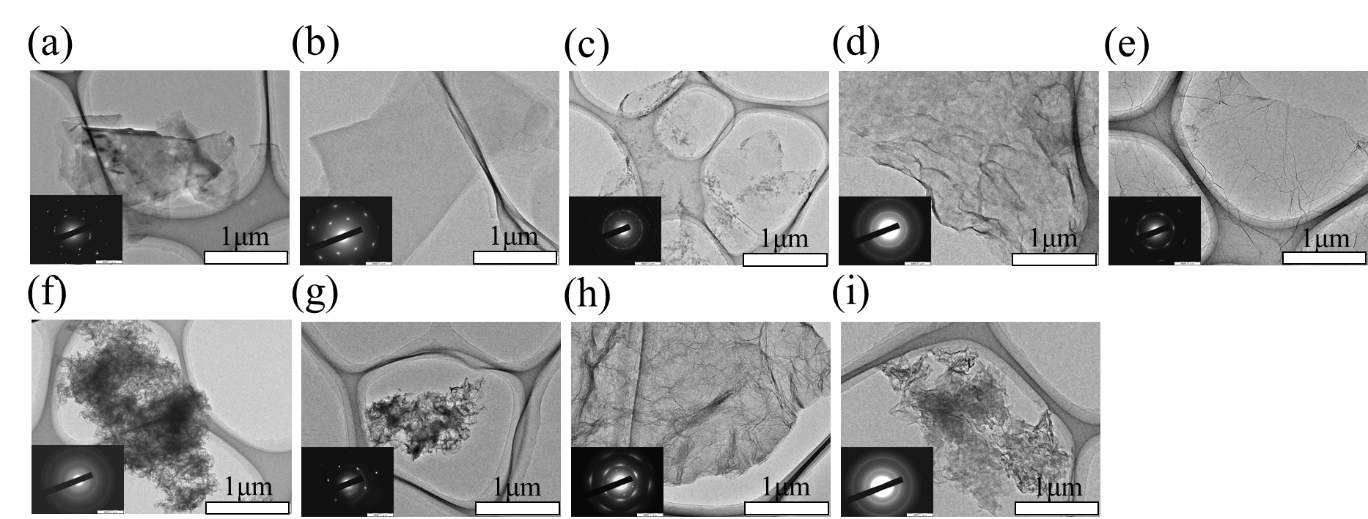


Figure S2 Bright-field TEM (BFTEM) images of (a) graphite, (b) graphene oxide, rGO (c) 0.5 h, (d) 1 h, (e) 2 h, (f) 4 h, (g) 6 h, (h) 8 h, and (i) 10 h samples. The insets are the corresponding selective-area electron diffraction patterns taken near the edge of the sheets for each sample.


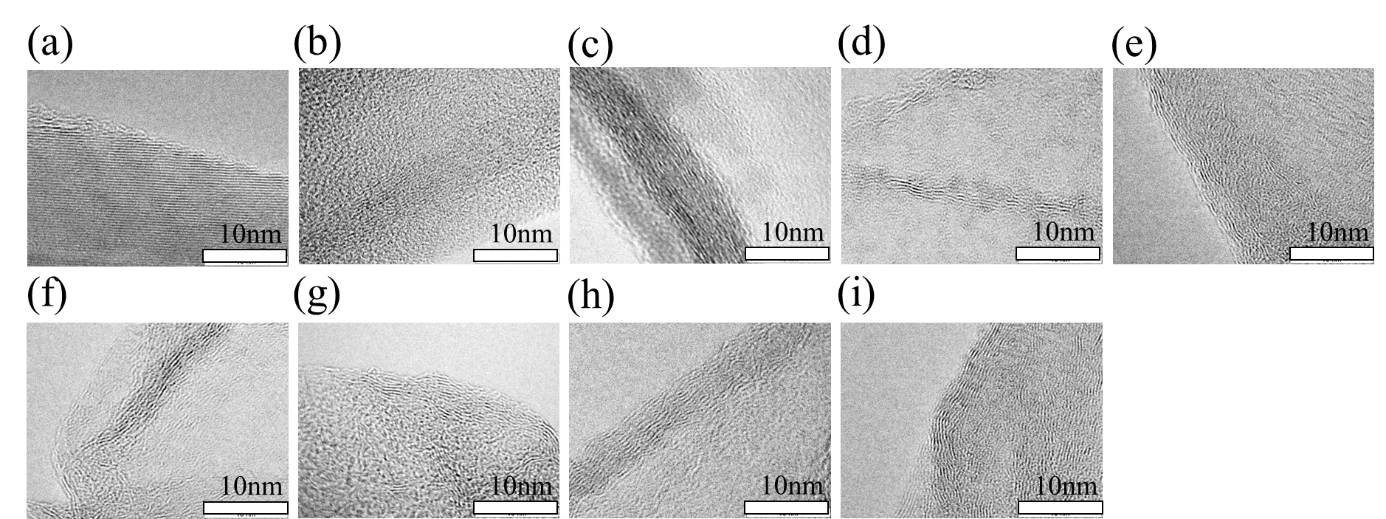


Figure S3 BFTEM images of (a) graphite, (b) graphene oxide, rGO (c) 0.5 h, (d) 1 h, (e) 2 h, (f) 4 h, (g) 6 h, (h) 8 h, and (i) 10 h samples taken near the edge of the sheets.

Table S1 The out-of-plane lattice parameters of raw graphite, GO, rGO 0.5 h, 1 h, 2 h, 4 h, 6 h, 8 h, and 10 h samples were calculated form XRD spectra.

| c-axis lattice parameters (nm) | |
| --- | --- |
| Graphite | 0.338 |
| GO | 0.810 |
| 0.5 h | 0.798 |
| 1 h | 0.746/0.350 |
| 2 h | 0.347 |
| 4 h | 0.357 |
| 6 h | 0.358 |
| 8 h | 0.357 |
| 10 h | 0.347 |


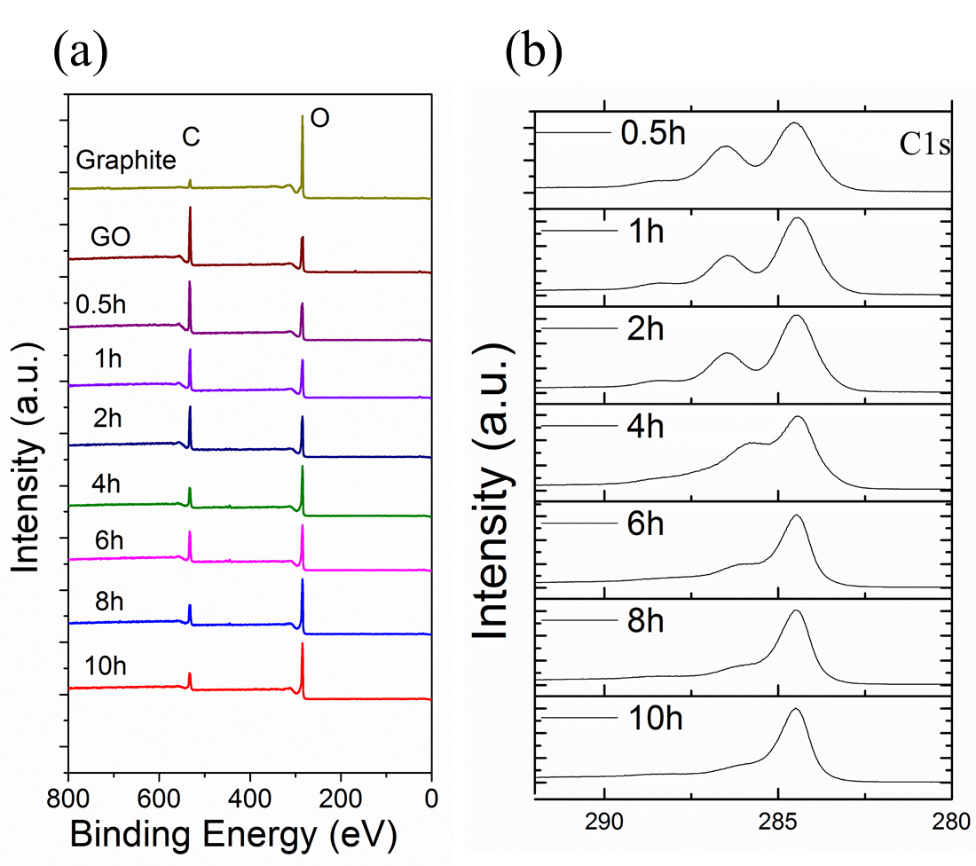


Figure S4 (a) XPS full spectra of graphite, graphene oxide, rGO 0.5 h, 1 h, 2 h, 4 h, 6 h, 8 h, and 10 h. (b) C1s high resolution XPS spectra of rGO 0.5 h, 1 h, 2 h, 4 h, 6 h, 8 h, and 10 h samples.


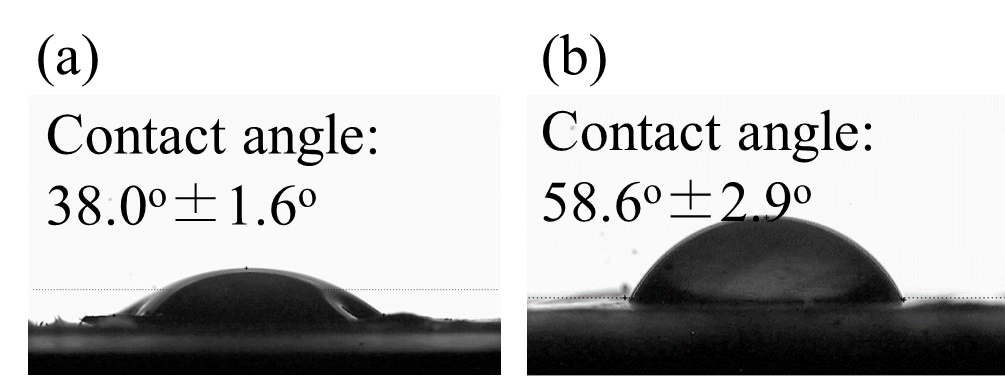


Figure S5 Contact angle measurement of (a) GO and (b) rGO 2 h.

**UV-visible spectroscopy analysis on rGO samples**

The progress of reduction was monitored using the UV-visible spectrometer by measuring the position of the highest absorption peak (λmax), as shown in Figure S6 (a). Noted that GO and its reduced products obtained at different time periods were dispersed in DI water at the same concentration via sonication. GO sample shows a λmax peak at 230 nm associated with π-π* transitions corresponding to the unoxidized C=C bonds and a shoulder peak at around 300 nm associated with n-π* transitions representing the C=O bonds. Upon reduction, the λmax peak has gradually red-shifted to 288 nm in rGO 8 h as a result of the restoration of C=C conjugation. Interestingly, for the samples reduced from 6 h, a doublet of the λmax peak was observed. It has been confirmed that this new peak does not stem from the DI water (see Figure S7). Instead, it could be due to the formation of smaller sp^2^ domains from breaking the C=C conjugation in rGO sheets under the prolonged reduction conditions. In rGO 10 h, the peak for C=C bond appears as a shoulder peak which is generally shown as a prominent peak. It could be due to the false information caused by the non-uniform dispersion of the solution as shown in Figure S6 (b). However, more qualitative measurement is required for detailed analysis. Nonetheless, the changes in absorption properties of GO and its reduced products were observed.


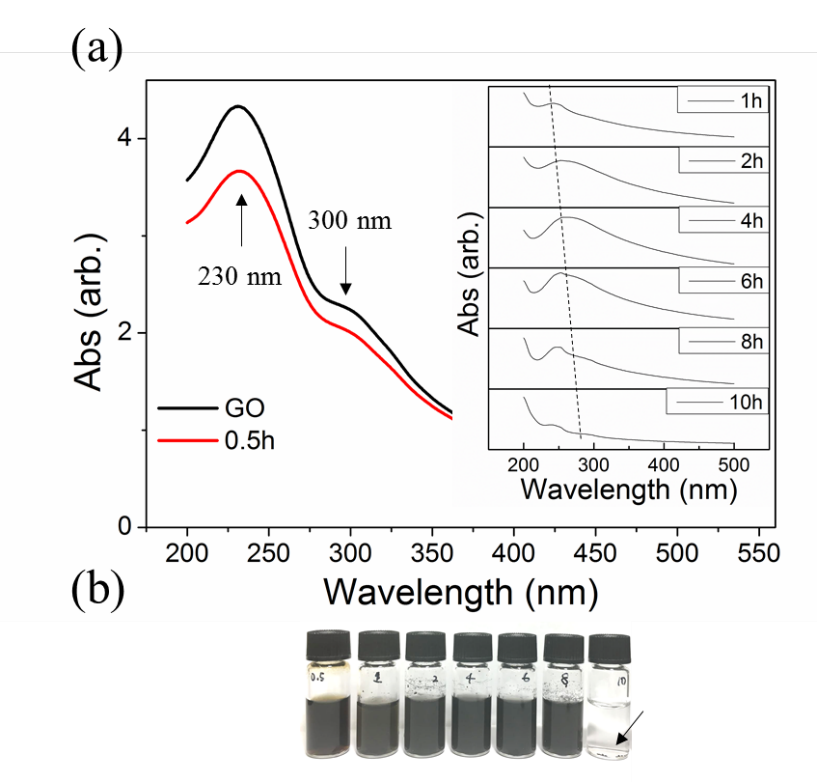


Figure S6 (a) UV-visible absorption for GO and a series rGO samples. (b) The dispersion of rGO 0.5 h, 1 h, 2 h, 4 h, 6 h, 8 h, and 10 h samples (from left to right).


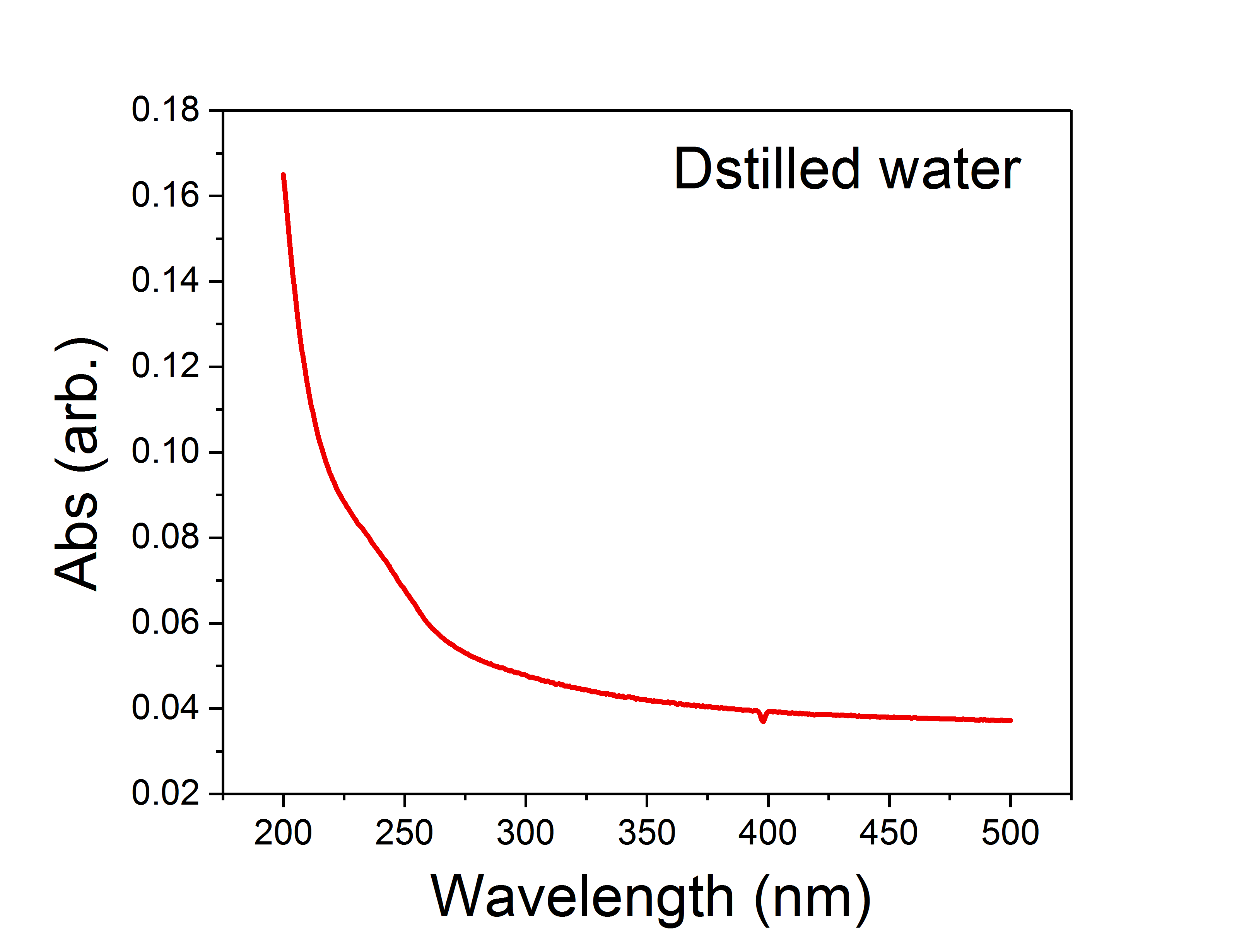


Figure S7 UV–visible optical absorption spectrum of distilled water.


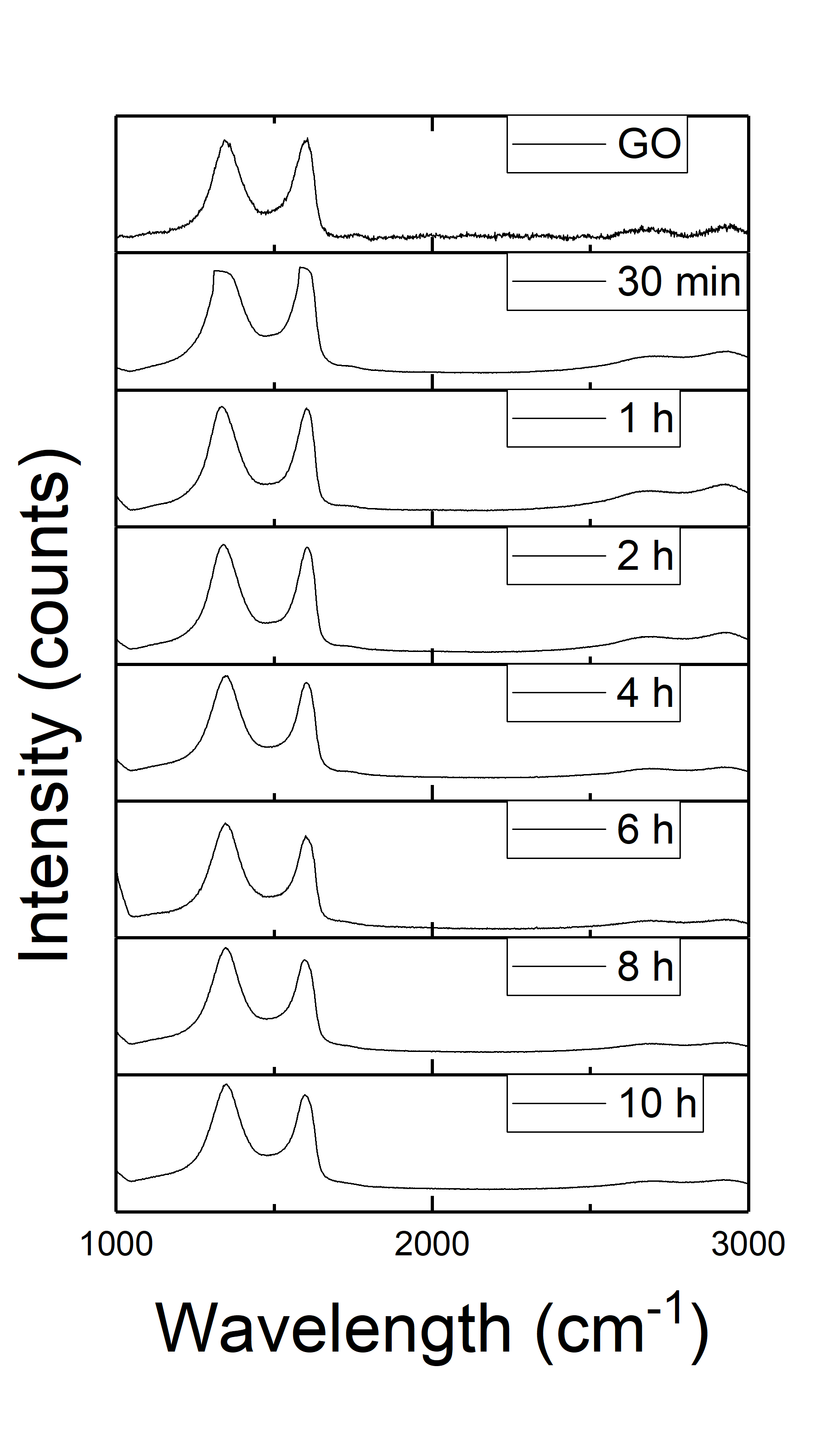


Figure S8 Raman spectra patterns of GO and rGO 0.5 h, 1 h, 2 h, 4 h, 6 h, 8 h, and 10 h samples.
